# Identification and Molecular Characterization of Spirurid Nematode Associated With Giraffe Skin Disease in Ruaha National Park, Tanzania

**DOI:** 10.1155/vmi/5053029

**Published:** 2025-05-20

**Authors:** J. Wanda, E. Mjingo, E. Mwega, J. Malago

**Affiliations:** ^1^Department of Veterinary Medicine and Public Health, College of Veterinary Medicine and Biomedical Sciences, Sokoine University of Agriculture, P.O. Box 3021, Morogoro, Tanzania; ^2^Tanzania Wildlife Research Institute, P.O. Box 661, Arusha, Tanzania; ^3^Department of Microbiology, Parasitology and Biotechnology, College of Veterinary Medicine and Biomedical Sciences, Sokoine University of Agriculture, P.O. Box 3019, Morogoro, Tanzania; ^4^Department of Veterinary Anatomy and Pathology, College of Veterinary Medicine and Biomedical Sciences, Sokoine University of Agriculture, P.O. Box 3016, Morogoro, Tanzania

**Keywords:** Giraffe Skin Disease, Masai giraffe, Tanzania, *Thelazia callipaeda*

## Abstract

Giraffe skin disease (GSD) is an emerging skin condition mainly affecting adult and subadult populations of free-ranging giraffe, including Masai giraffe *(Giraffa tippelskirchi)* in the southern and northern protected areas (PAs) in Tanzania. Even though GSD has been described in Tanzania, the specific involvement of the spirurid nematode as the underlying cause of the lesions has been suggested but not definitively established. This cross-sectional study aimed to characterize spirurid nematodes associated with GSD lesions by analyzing 10 skin biopsies collected between October and November 2022. Histopathological examination revealed spirurid larvae in 6 out of 10 skin biopsies, with variable numbers found within the dermis, alongside the presence of clear vacuoles, eosinophils, neutrophils, lymphocytes, and fibrous connective tissue. Three biopsies tested positive for the 28S rDNA using a conventional polymerase chain reaction and provided a sequence of *Thelazia* spp. submitted in GenBank (accession no. 0R466406). The phylogenetic tree showed close similarity to *T. callipaeda* (99.11%, accession no. MF953480, and 99.38% accession no. MK214873, respectively). This study has shown the presence of the Spirurida, Thelaziidae worm in skin lesions of Masai giraffes affected by GSD. Specifically, this research documents the occurrence of *T. callipaeda* in the skin lesions providing valuable insight into parasitic involvement. Despite these findings, the mechanisms by which the nematode is transmitted to the giraffe's skin remain unknown. Further study is required to understand the impact of *Thelazia callipaeda* on both GSD and non-GSD giraffes to better understand the potential cause of GSD.

## 1. Introduction

Giraffes (*Giraffa* spp.) are the tallest animals of the African savannah and are widespread in small isolated populations, with an estimated population of approximately 117,173 individuals globally [1]. The Masai giraffe (*G. tippelskirchi*), which is native to East Africa, is common throughout Tanzania and is also found in central and southern Kenya; however, the species population has declined by 40% in the three decades, resulting in an estimated population of about 45,402 individuals, therefore, listed as an endangered species [2]. Like many other wildlife species in Africa, the Masai giraffe is threatened by diseases, human-wildlife conflict, habitat loss, and poaching [3, 4]. These threats affect the giraffe's population and negatively impact the economic revenues from tourism [5]. Emerging diseases pose a substantial risk to wildlife, especially in populations already facing other pressures such as habitat loss and poaching [6]. They include parasitic diseases that may substantially affect wildlife population dynamics and signify a critical issue in the conservation of endangered species [7].

The remaining isolated populations of Masai giraffe are threatened by a condition referred to as giraffe skin disease (GSD) first described in Ruaha National Park (RNP), southern Tanzania, with a prevalence of 85% [3, 8]. In Tanzania, GSD manifests as small skin nodules of about 2-3 cm in diameter with raised hairs that later coalesce into large round or oval patches of 10–16 cm in diameter; the skin hardens, dries out, wrinkles, and cracks resulting in oozing pus and mostly affects the forelimb's medial side in adult and subadult Masai giraffes [8, 9]. Surveillance studies in other protected areas (PAs) have shown that GSD spreads from the southern region to the northern PAs of Tarangire, Manyara, and Serengeti National Parks [10]. A cross-sectional survey conducted in the Tarangire–Manyara ecosystem showed that 69% of the Masai giraffes were affected with skin lesions, suggesting a likely association between fungal infestation and the occurrence of GSD [11] Indeed, the authors in [8] suggested the involvement of spirurid nematode in GSD and the possibility of secondary infection of fungi and bacteria. Recently, the authors in [12] found that nematodes are morphologically and genetically similar to the novel ones with close identity to *Stephanofilaria* spp and *Brugia malayi* in this condition. However, none of these etiologies has yet been confirmed as the main cause of GSD. Therefore, the current study aimed to identify and characterize spirurid nematodes of skin lesions observed in Masai giraffes.

## 2. Materials and Methods

### 2.1. Study Area

**Figure 1:**
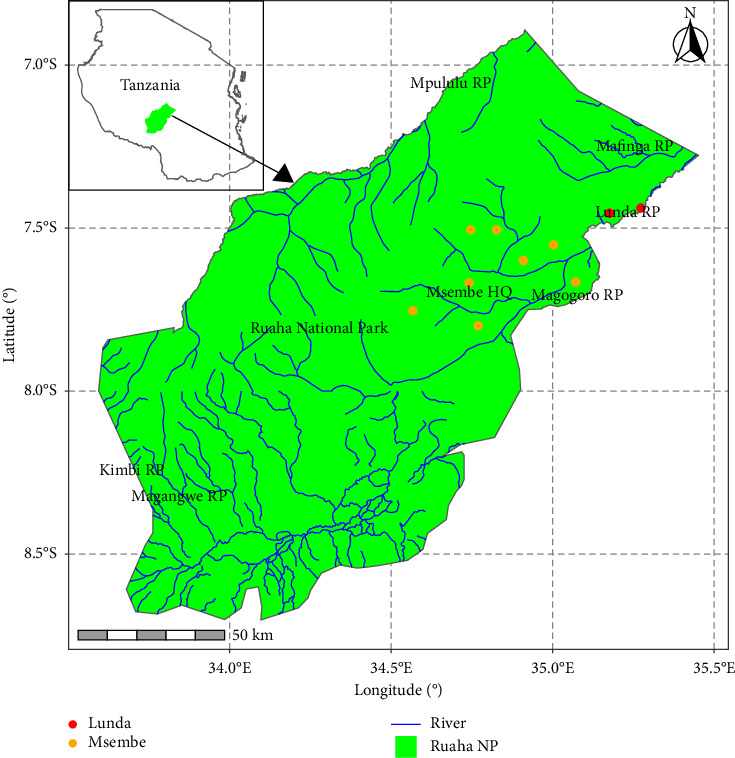
Map of Tanzania showing the position of Ruaha National Park and sampled site highlighted in red and yellow spots.

The study utilized samples collected from RNP, the second-largest national park in Tanzania, as illustrated in Figure 1. Covering an area of 20,226 km^2^, RNP is situated in the southern highlands of Tanzania, approximately 130 km west of Iringa town, between 7°30′S and 8°00′S latitude and 33°50′E and 35°25′E longitude together with adjacent game reserves, such as Rungwa Game Reserve, Kizigo Game Reserve, Muhesi Game Reserve, and Lunda-Mkwambi Game Controlled Area, which forms the Greater Ruaha–Rungwa ecosystem (GRRE), spanning approximately 45,000 km^2^. The GRRE features diverse habitats including savannah woodlands, swamps, and riverine areas, leading to a rich variety of fauna and flora. The RNP is bounded by two significant rivers, the Mzombe River to the north and the Great Ruaha River to the south, which serve as vital water sources for the park's wildlife species.

### 2.2. Animal Immobilization and Sample Collection

**Figure 2:**
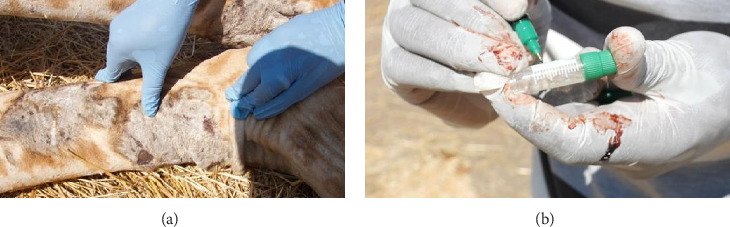
(a) Gross skin lesions associated with GSD were observed on the fore limbs of an anesthetized Masai giraffe, characterized with alopecia, crusting, and hyperkeratosis. (b) Skin biopsy collected from the lesion.

A giraffe was immobilized with a total dose of 16–18 mg etorphine hydrochloride (Immobilon, Veta Pharma Ltd), remotely using 2cc darts (Pneu-Dart, Incl.), shot from a Model 196 Pneu-Dart cartridge-fired projector at a reachable distance of about 15–20 m targeting the shoulder or thigh. A total of five Masai giraffes of each sex were purposively examined and sampled for the presence of GSD with consideration given to the severity and site of the lesion.  Figure 2. Whole blood and skin biopsy were collected aseptically from the skin lesion using a punch biopsy of 3 mm [13]. Giraffe was subsequently reversed through an intravenous injection using diprenorphine hydrochloride (M5050), with a total dosage of 35–40 mg. All 10 skin biopsies were collected during fitting Global Positioning System (GPS) tags conducted in RNP in October-November 2022. The samples were preserved in 10% neutral buffered formalin (10% NBF) for histopathology analysis. Each skin biopsy from each clinical case was duplicated and preserved in RNAlater (Sigma-Aldrich Co, St. Louis, MO, USA) following the manufacturer's instructions and then stored at −80°C. The preserved samples were transported to the Pathology and Microbiology Laboratories at the College of Veterinary Medicine and Biomedical Sciences at the Sokoine University of Agriculture (SUA) for analysis.

### 2.3. Histopathological Analysis of Skin Biopsies

The collected skin biopsies were fixed in 10% NBF, followed by standard processing procedures. In summary, the procedure included dehydrating the tissues in a series of ethanol solutions (70%, 95%, 95%, and absolute), and clearing in xylene, embedded in paraffin wax. Tissues were sectioned at a size of 4 *μ*m using a microtome (Baird and Tatlock London LTD, Chadwell Health, Essex, England., Ref. no. 368065/2), and then stained with hematoxylin and eosin (H&E) and affixed with permanent optical grade glue to ensure adhesion. Tissue cellular changes and parasite identification were achieved using a binocular light microscope (Olympus Corporation, UDO3, S/N 9M11951, Tokyo, Japan).

### 2.4. DNA Extraction

DNA was extracted from six positive samples out of 10 skin biopsies using a commercial kit (QuickDNA Universal Kit, South Africa) by Zymo Research, following the manufacturer's instructions. In brief, 0.025 g of homogenous skin tissue suspended in equal volumes of 95 μL of water and tissue buffer was digested with 10 μL proteinase K following incubation at 55°C for 3 h. The digested tissue was centrifuged at 12, 000 × g for 1 min and 200 μL of the supernatant was recovered in a clean 1.5 mL Eppendorf tube. To bind the extracted DNA, 400 μL of the genomic binding buffer was added to 200 μL of the supernatant. This mixture was transferred to a Zymo-spin IIC-XL column and centrifuged at 12, 000 × g for 1 min. DNA purification involved a series of washing steps using wash buffers. Bound DNA was recovered from the columns using an elution buffer. Before storage, the extracted DNA was subjected to a NanoDrop spectrophotometer for concentration measurement. The final DNA extract was subsequently refrigerated at −20°C awaiting further analysis.

### 2.5. DNA Amplification

The extracted DNA was amplified using a conventional polymerase chain reaction (cPCR), targeting 765 bp of 28S rDNA of the nematode worms as previously detailed [14]. Amplification was performed with a forward primer (5′-GCGGAGGAAAAGAAACTAA-3′) and a reverse primer (5′-ATCCGTGTTTCAA GACGGG-3′). The PCR reaction mixture (25 μL) comprised 12.5 μL of OneTaq 2x master mix, 0.5 μL of each forward and reverse primer, 7.5 μL of nuclease-free water, and 4 μL of purified DNA. The amplification process began with an initial denaturation step at 95°C for 5 min, followed by 35 cycles. Each cycle consisted of denaturation at 94°C for 1 min, annealing at 50°C for 30 s, and extension at 72°C for 1 min. The final extension was carried out at 72°C for 7 min. The PCR analysis was performed using the ProFlex PCR system. The resulting PCR products were analyzed by electrophoresis on a 1.5% agarose gel with a 100 bp DNA ladder. The results were visualized using a UV transilluminator and by employing a gel doc (Vilber format machine). PCR products were subsequently purified according to the manufacturer's instructions utilizing the GeneJET Genomic DNA Purification Kit (Thermo Fisher Scientific). The resulting purified PCR products were submitted for Sanger sequencing to verify the presence of spirurid nematodes.

### 2.6. Sequence and Phylogenetic Tree

Purified PCR products were sequenced using both the forward and reverse strands at a commercial sequencing company (Macrogen Laboratory, Korea). The spirurid nematode sequences obtained were aligned using the ClustalW algorithm in MEGA V.11 [15]. These aligned sequences were compared for similarity with sequences stored in GenBank. The phylogenetic analysis was constructed using the neighbor-joining method [16], and the confidence of the tree's branching pattern was estimated through bootstrap analysis with (1000) replications [17]. The resulting tree was accurately scaled, with branch lengths denoting evolutionary distance. Distances were calculated using the Kimura 2-parameter method [18] and were expressed as the number of base substitutions per site. Pairwise deletion was used to remove ambiguous positions within each sequence pair, and all analyses were performed in MEGA V.11 [15].

### 2.7. Research Permit

The research was reviewed and cleared by the Tanzania Commission of Science and Technology and Tanzania Wildlife Research Institute permit number: 2020-192-NA-2020-70.

## 3. Results

### 3.1. Histopathology

**Figure 3:**
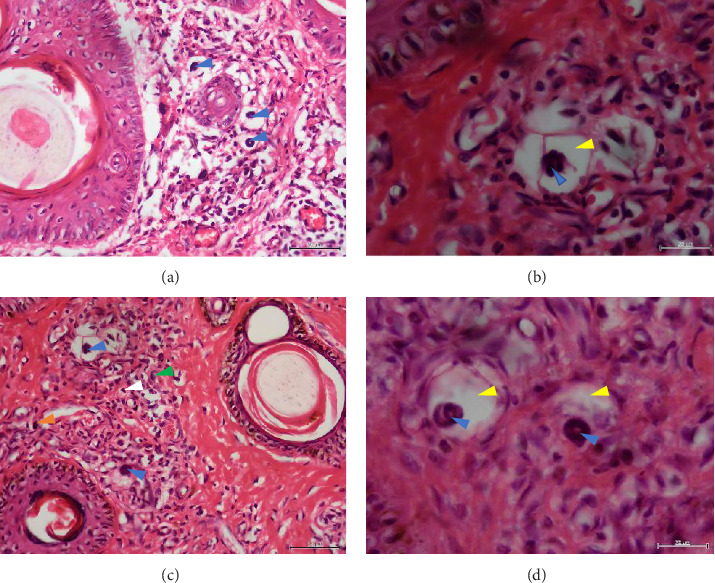
(a–d) Nematodes localized within the dermis and outer sheath of the hair follicles of Masai Giraffe skin (blue arrows). (c) Inflammatory cell infiltration: eosinophils (green arrow), lymphocytes (white arrow), and macrophages (orange arrow). (b and d) Clear vacuoles around the nematode (yellow arrows). H&E, X40 objective lens (a and c). High magnification X100 objective lens (b and d).

Histopathological examination of 6 out of 10 tissue biopsies unveiled variable numbers of spirurid larvae localized at the dermis layer of skin lesions and inflammatory cell infiltration, such as eosinophils, neutrophils, and lymphocytes. Remarkably, clear vacuoles were observed, indicating cellular degeneration or damage. Tissue dermatitis and an increased presence of fibroblastic connective tissue were also observed Figure 3.

### 3.2. PCR Sequence and Phylogenic Analysis

**Figure 4:**
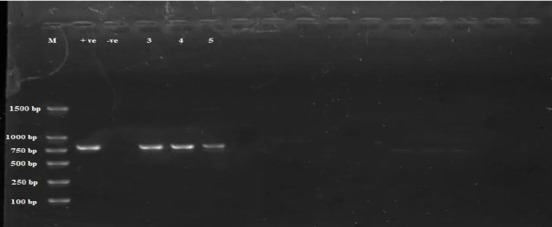
Gel electrophoresis image shows PCR products of the 28S rDNA. Lane M represents a 100 bp marker, and Lanes 1 and 2 are positive and negative controls, respectively. Lanes 3–5 display positive samples at an expected band size of 765 bp.

Subsequent analysis involved the utilization of cPCR that targeted 28S rDNA. Among the six samples evaluated, three were positive (Figure 4).

**Figure 5:**
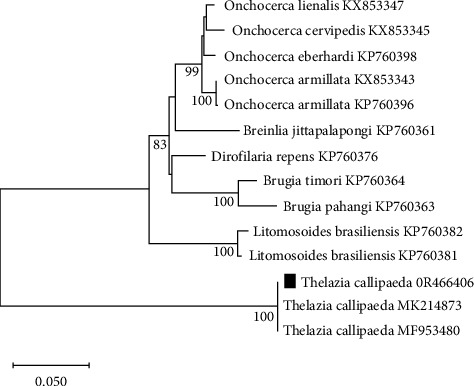
Illustration of the phylogenetic tree constructed from partial sequences of spirurid nematode isolates encompassing 765 bp. The tree is compared with other spirurid nematodes available in GenBank.

The sequence generated was deposited in GenBank (accession no. 0R466406). The partial 28S rDNA sequences of *Thelazia* spp. showed a maximum similarity to one well-supported clade with a bootstrap support value of 100% (Figure 5). The closest match was *Thelazia callipaeda* (99.11%, accession no. MF953480, and 99.38%, accession no. MK214873, respectively).

## 4. Discussion

In Tanzania, GSD–associated lesions have been putatively related to spirurid nematodes [19]. These results strongly align with previous investigations, substantiating the role of spirurid nematodes in causing GSD [3]. Histopathological examination of the skin biopsies showed varying numbers of these spirurid larvae localized within the dermis layer of skin lesions (Figures 3(a), 3(b), 3(c), and 3(d)). This localization pattern suggests a specific affinity of the spirurid worms for the dermal tissues, possibly indicating an adaptation to this microenvironment and their involvement in the pathogenesis of GSD. Furthermore, as illustrated in Figure 3(c), the presence of inflammatory cell infiltration, comprising eosinophils, neutrophils, and lymphocytes, indicates an active immune response against the parasitic infection. Eosinophils are particularly indicative of helminth infection, suggesting an allergic or hypersensitivity reaction against the invading spirurid larvae. Neutrophils are the body's first line of defense against bacterial infections resulting from the skin damage caused by the worms [20]. The observed clear vacuoles within the affected tissues (Figures 3(b) and 3(d)) are indicative of cellular degeneration or damage, suggesting that the presence of the spirurid larvae has led to the pathological changes in the host tissues, and this damage could result from the mechanical action of the worms during penetration or migration within the dermis layer. Furthermore, the identification of tissue dermatitis, characterized by inflammation of the skin tissues, highlights the pathological consequences of the parasitic infection.

The sequences of the purified PCR products from the detected spirurid nematode revealed high similarity percentages, suggesting their close relation with *Thelazia callipaeda*, a known species from the *Thelazia* genus.

A parasitic roundworm with zoonotic potential is known to infect both the eyes of animals and humans and is commonly referred to as the “oriental eyeworm” [21]. This parasitic organism is transmitted by secretophagous flies and affects humans and carnivores [22]. These findings represent a *Thelazia* spp., a worm closely related to *T. callipaeda* in wildlife in Tanzania, and remarkably, the first report of this nematode found in the skin lesion of the Masai giraffe (*Giraffa tippelskirchi*). Despite confirming the presence of the nematode, the specific mechanism by which the Masai giraffe acquired the eyeworm infection in the skin lesion remains unclear. However, several factors could contribute such as transmission pathways of *Thelazia* spp. from its natural hosts, behavior patterns such as feeding habits, interactions with other wildlife species, environmental factors, human-induced changes, and genetic mutation of *Thelazia* spp.

*T. callipaeda* has been documented in countries across Europe and Asia, where carnivores such as *Canis lupus*, *Felis catus*, and *Vulpes vulpes* play a vital role in the eyeworm species' sylvatic cycle [23, 24]. This suggests a species-specific adaptation to this particular ecosystem. There is also a possibility that this species has evolved from older, nonpathogenic worms and has become prevalent in the environment coupled with the greater mobility of animals across the park leading to the proliferation of diseases in regions previously unaffected.

GSD has been reported with clinical manifestations similar to those observed in Uganda, where filarial sequences were identified [25]. The skin biopsy approach used in our study differs from the skin scrapes utilized in the Uganda study, possibly due to the predilection site of the parasite [26]. Interestingly, a study conducted in the Republic of South Africa identified *Stephanofilaria* nematodes from the skin of a hippopotamus to have morphological similarities to *Thelazia* spp. Moreover, it suggested that the isolated *Stephanofilaria* can be named *Stephanofilaria thelaziidae* spp. [27]. Furthermore, PAs harbor numerous sylvatic species (e.g., *Canius aureus* and *Vulpes vulpes*), which are themselves capable of being reservoirs for *T. callipaeda* [28, 29]. Even though the infectious agent has been identified, this study implies that wild carnivores could act as natural reservoirs for *T. callipaeda* in PAs.

Tanzania has a large population of *Panthera leo* compared to other countries across Africa, which may increase the likelihood of *T. callipaeda* being established [30]. These findings suggest the possibility of the existence of a sylvatic lifecycle of *T. callipaeda* in wild carnivores. Other studies have utilized 28S and revealed phylogenetic evidence between *Stephanofilaria stilesi* and *T. callipaeda,* highlighting the importance of reevaluating their existing taxonomy with a possible reallocation of *Stephanofilaria stilesi* from Filarioidea to Thelazioidea [26]. This study provides evidence that the spirurid nematode, *Thelazia* spp., was detected in 6 out of 10 GSD lesions, suggesting it may be associated with the pathogenesis of GSD. In addition, this study not only underscores the necessity of further investigation focusing on the molecular characterization by utilizing next-generation sequence techniques and epidemiological aspects of this nematode in GSD–affected giraffe populations but also sheds light on the potential pathogenic role of *T. callipaeda* previously not reported in Tanzania.

## 5. Conclusion

In conclusion, this study provides the evidence of *T. callipaeda* worm, a type of spirurid nematode within skin lesions affected by GSD. However, the exact mechanisms of transmission of the nematode to the giraffe's skin remain unclear. Further research is needed to understand the epidemiology and transmission dynamics of this worm to giraffes. We recommend that further research by conducting genomics studies examining a broader range of samples from both affected and unaffected animals would provide a decisive understanding of the potential cause of GSD.

## Data Availability

The data used to support this finding are available from the corresponding author upon request.
